# Biofunctional Understanding and Conceptual Control: Searching for Systematic Consensus in Systemic Cohesion

**DOI:** 10.3389/fpsyg.2017.01702

**Published:** 2017-10-24

**Authors:** Asghar Iran-Nejad, Fareed Bordbar

**Affiliations:** Department of Educational Studies in Psychology, Research Methodology, and Counseling, The University of Alabama, Tuscaloosa, AL, United States

**Keywords:** biofunctional understanding, declarative fact-seeking, procedural knowhow, embodiment science, spiral of biofunctional understanding, systematic observation, systematic consensus, unobservable systemic cohesion

## Abstract

For first generation scientists after the cognitive revolution, knowers were in active control over all (stages of) information processing. Then, following a decade of transition shaped by intense controversy, embodied cognition emerged and suggested sources of control other than those implied by metaphysical information processing. With a thematic focus on embodiment science and an eye toward systematic consensus in systemic cohesion, the present study explores the roles of biofunctional and conceptual control processes in the wholetheme spiral of biofunctional understanding (see Iran-Nejad and Irannejad, 2017b, Figure 1). According to this spiral, each of the two kinds of understanding has its own unique set of knower control processes. For conceptual understanding (CU), knowers have deliberate attention-allocation control over their first-person “knowthat” and “knowhow” content combined as mutually coherent corequisites. For biofunctional understanding (BU), knowers have attention-allocation control only over their knowthat content but knowhow control content is ordinarily conspicuously absent. To test the hypothesis of differences in the manner of control between CU and BU, participants in two experiments read identical-format statements for internal consistency, as response time was recorded. The results of Experiment 1 supported the hypothesis of differences in the manner of control between the two types of control processes; and Experiment 2 confirmed the results of Experiment 1. These findings are discussed in terms of the predicted differences between BU and CU control processes, their roles in regulating the physically unobservable flow of systemic cohesion in the wholetheme spiral, and a proposal for systematic consensus in systemic cohesion to serve as the second guiding principle in biofunctional embodiment science next to physical science’s first guiding principle of systematic observation.

## Introduction

### The Myth of the Knowledge Stored in Connections

In a panel discussion entitled “The computational conception of mind” with Gilbert Harman, John Haugeland, Jay McClelland, Allen Newell, Dana S. Scott, and Zenon Pylyshyn as participants, the moderator, Scott (1990) asked, *Will there be a theory of comprehension?* The answer to be sought by the audience was hidden in the moderator’s statement “In our view, the implicit knowledge is stored in connections among simple processing units organized into networks” (p. 39). This excerpt is widely circulated in unmistakably similar words throughout the community of the second generation cognitivist, especially, the literature on parallel distributed processing (PDP), a well-known predecessor to embodied cognition (see Harnad, 1990; Iran-Nejad and Homaifar, 2000).

For more than a decade after the cognitive revolution, short-term control processes and long-term storage architectures dominated the field of first-generation cognition (Neisser, 1967; Atkinson and Shiffrin, 1968). Mind connections, frames, and hierarchies ranged from the most concrete sensory to the most abstract conceptual levels (Rumelhart and Ortony, 1977; Rumelhart, 1980). Network metaphors were everywhere representing unobservable mind connections in concept maps, semantic spaces, and memory taxonomies for saving content inside knowers. The mechanistic vernacular of the first-generation cognition, mostly metaphysical in nature, inspired by the computer-program analogy (Neisser, 1967), soon faced challenges from critics (Bransford and Johnson, 1972; Iran-Nejad, 1980/1987), could not stay free from trouble (Jenkins, 1974; Bransford et al., 1977; Iran-Nejad and Ortony, 1982; Shulman, 1999), and, before long, its pioneers began scrambling for replacement alternatives.

Metaphysical information processing scientists found their cues in spatial and computer metaphors (Roediger, 1980); and were inclined accordingly to mechanize human information processing (e.g., spreading activation) and humanize mechanical architectures, e.g., neural networks (Collins and Loftus, 1975; Beers, 1987; Smolensky, 1987). As a result, when information processing controls and architectures were still at the peak of their popularity, Neisser (1976), who wrote the original book on soft cognitive psychology less than a decade earlier (Neisser, 1967), lamented that information-processing and storage constructs had prestige and momentum but their computer-inspired control processes and structures ran contrary to the human nature (Iran-Nejad and Winsler, 2000). Neisser (1976) himself abandoned the computer metaphor in a hurry and turned first to ecological psychology (Gibson, 1966) and then to biology in search of a more natural human cognition (Neisser, 1987, 1994; Neisser and Winograd, 1988; Neisser and Jopling, 1997). Nevertheless, even today, articles, books, and even entire journals keep spreading Neisser’s (1967) metaphysical footsteps in leaps and bounds across this planet (Khemlani et al., 2014). How could one answer Dana S. Scott’s question about comprehension or make room for human understanding in this unlikely terrain?

As suggested by the above discussion panel, the momentum Neisser (1976) saw at the expense of human nature kept rising for a few more years (Rumelhart, 1975, 1976; Anderson, 1977; Rumelhart and Ortony, 1977; Schneider and Shiffrin, 1977; Shiffrin and Schneider, 1977; Anderson et al., 1978; Brewer and Lichtenstein, 1981; Brewer and Nakamura, 1984; Brewer, 2000). Then, it faced stiff resistance from supporters of human cognition (Black and Wilensky, 1979; Iran-Nejad, 1980/1987; Thorndyke and Yekovich, 1980; Iran-Nejad et al., 1981/1984; Alba and Hasher, 1983; Beers, 1987). During this transition period, many authors worked on connectionist, computational, and spreading-activation networks or similar metaphors that honored, in the words of Beers (1987), “the major tenets of schema theory by making them conform more clearly to an explicitly ecological perspective relating human understanding to the environment—social and otherwise—in which it takes place” (p. 376). Nevertheless, the reality of the human nature Neisser (1967) had first abandoned and then sought (Neisser, 1976) went his way neither time.

### The Myths of Vegetative Organs and Smart Connections

In the 1990s, the second generation of the science of cognition arrived in a hurry; and, in a short decade or two, embodied cognition swept the planet (Wilson, 2002; Adams, 2010), filling rapidly the post-transition void for a new mainstream field of cognition we own today. However, the unsystematic diversity of these embodied cognition networks, as evident in the titles and texts of the related literature reviews, must have been overwhelming. For example, in characterizing this period, Kiverstein (2012), among others (Wilson, 2002; Gärtner, 2011, 2013), asked exactly what researchers had in mind when they claimed that cognition is embodied. He came back empty-handed and described the diverse movement in terms of four cold Es (embodied, embedded, extended, enacted) and one hot A (affective). Incidentally, Kiverstein left out one more E (expanded) to account for Clark’s (2008) *Supersizing the mind* apparently by adding more body to the head and more head to the body. Kiverstein traced the origin of the diverse field of embodied cognition uniformly to the *Embodied mind* (Varela et al., 1991) that “sought to bring about nothing short of a paradigm change” (p. 741). Others suggested that the embodied mind perspective may be a revolution away from the first generation disembodied cognition as well as one from the second generation embodied cognition, a view with which we tend to agree (see Gärtner, 2013). Embodied cognition is. as Kiverstein (2012) suggested, about how cold and hot cognitions are embodied, how they are tacitly embedded in smart network connections, how they extend storage to external media, and how they enact downloads to the body and the world or uploads back into the head and its supersized networks of smart connections, all of which means knowledge is stored in smart connections without at least for now a trace of D. S. Scott’s comprehension, not to mention understanding. Embodied mind researchers, by contrast, lean heavily in favor of the biofunctional wisdom of the biological body to ground the contemplative wisdom of the human conceptual understanding (Rosch, 2000, 2001) and find the mechanistic embodied cognition hard on the palate.

### Purpose

The focus of embodied cognition during the second generation cognition continued to be on knowledge and never on understanding. The overall concern of the present study is with biofunctional understanding and in that special sense with the embodied mind (Lakoff and Johnson, 1980; Posner et al., 1982; Shulman, 1991, 2002; Johnson, 2015). To be sure, understanding was sought by embodied cognition researchers without the inclination to say or pretension to know the word as they looked for revolutions in expressions like symbol-grounding (Harnad, 1990), PDP connectionism (McClelland and Rumelhart, 1986; Rumelhart and McClelland, 1986; Rumelhart et al., 1986) or deep processing (Craik and Lockhart, 1972; Craik and Tulving, 1972). A closer look at the work of embodied cognition pioneers in neuroscience like Edelman (2006) and cognition such as Clark (2008) revealed that they used the concept of understanding 51 and 71 times, respectively, but invariably in the instrumental sense of the term for making sense of other things but never as a construct under investigation in its own right—like knowledge. Perhaps for these embodied cognition scientists, the best that the vegetative body could do would be to save in its intelligent neural networks its own content knowledge.

### Understanding in the Transition Era

In the heat of the transition period, two lines of research in cognitive psychology addressed understanding directly, both rising, in part, to challenge the myth that knowers needed abstract deep structures to reach the realm of understanding (see, however, Shulman, 1984, 1999). One of these came in a critique by Black and Wilensky (1979) of Rumelhart’s (1975) first generation story grammar (Wilensky, 1983). Commenting on Rumelhart’s pioneering claim that knowers had to employ deep structures to understand stories, these researchers reasoned that deep story structures presupposed rather than caused understanding. The second line of research came in the form of the biological embodiment of understanding and the straightforward assumptions that understanding is the special and unique function of the biofunctional wisdom in the nervous system as the one and only direct, necessary, and sufficient prerequisite for understanding, just as respiration was the special function of the biological activity in the respiratory system as the one and only direct, necessary, and sufficient condition for breathing (Iran-Nejad, 1980/1987; Iran-Nejad and Ortony, 1984; Iran-Nejad and Irannejad, 2017b). The biofunctional view of understanding is that biological systems, subsystems, and microsystems (i.e., neurons) take their turns to be the immediate and direct production site for the intellectual performance that their specialty prescribes. Therefore, far from what is implied by the botanical metaphor of vegetative organs often applied to them, the biological systems of the body are miraculous contributing sources, each in its own marvelously consensual way, to a very special type of wisdom aptly, we believe, called *biofunctional understanding* (Johnson, 2015).

### Embodying Understanding One Metaphor at a Time with Both Hands Tied in the Back

The transition era provided scarce ground for the kind of evidence, theory, and methodology about biofunctional understanding that is available worldwide today (e.g., Iran-Nejad, 2000; Ziemke et al., 2004; Borghi et al., 2013; Ghorbani et al., 2014; Alverson, 2015; Jin et al., 2015, 2016; Johnson, 2015; Billing et al., 2016; Caligiore et al., 2016; Soylu, 2016; Thill and Twomey, 2016). Therefore, early biofunctional theorizing had to scrape for embodiment one metaphor at a time, just as one had to struggle breathlessly against the downhill current of prestigious metaphysical cognitive psychology (Iran-Nejad, 1980/1987; Iran-Nejad and Irannejad, 2017b). Biological metaphors were shunned vehemently and rejected out of hand by editors, reviewers, and readers alike. Mechanical metaphors were more likely to be allowed; but seldom grabbed attention in the metaphysical world of cognition. Embodied metaphors like color-coded lightbulbs—to represent dynamic diversity in unity and cohesive unity in diversity—were used for distributed constellations of firing neurons, blinking traffic arrows were used for their diverse-content sensemaking behavior, and momentary constellation firing was used to represent the multiple-source nature of the dynamic sensemaking process (Iran-Nejad, 1980/1987, 1984; Iran-Nejad et al., 1981/1984; Iran-Nejad and Ortony, 1984). Nevertheless, the sharply vivid metaphors notwithstanding, the experience was nothing less than swimming against a sharply downhill current.

The vivid analogy of the manual camera was used to protect biofunctional theory against the myth of saved prior knowledge—in the form of deep structures or otherwise. The prior knowledge hypothesis assumed that past knowledge is inevitable for new learning to occur. The biofunctional theory explicitly disavowed and abandoned this assumption and used the analogy of the manual camera to show how understanding was possible without saved prior knowledge (Iran-Nejad, 1980/1987). However, the assumption of saved prior knowledge was so deeply entrenched that it kept appearing in the reviews of embodied cognition three decades later. Consider the title of the review by Gärtner (2013): “Cognition, knowing and learning in the flesh: six views on embodied knowing in organization studies.” This title strongly implies that embodied cognition meant knowledge was saved in the flesh of the body. In fact, the assumption of saved prior knowledge, inevitable for metaphysical theories, is contrary to both the letter and the spirit of biofunctional theory.

The analogy of the manual camera was desperately used in the late 1970s to show that the saved prior knowledge assumption was unnecessary for biofunctional theory (see below). To picture external objects, a mechanical camera needed no internal blueprints for them and, in fact, such blueprints and their hegemonic character would get in the way of accommodating the ubiquitous phenomena of cohesive unity in diversity and productive diversity in unity. To picture a dog, a mechanical camera needed to know neither a disembodied internal template to match against the abstract shape of the dog, as assumed by first-generation cognition, nor an embodied internal statue to match against the body of the dog, as suggested by second generation embodied cognition. To picture a dog, the mechanical camera needed only its own physical hardware and no saved prior knowledge at all. Dynamic biofunctional embodiment of understanding was proposed to counter the theory of embodiment as saved prior knowledge in the body or in the head and to get rid of the assumption altogether. Neither the mechanically crude wisdom in the manual camera nor the organically sophisticated wisdom in the biofunctional body were the wisdom of saved prior knowledge. According to biofunctional theory, biological flesh had the capacity to create knowledge on demand but no capacity to save and retrieve it whatsoever. That much must have been driven home for the proponents of the saved prior knowledge theory in the 1970s because enough of them rapidly packed their tools and abandoned their so-called structural schema theories and began scrambling for replacement alternatives (e.g., Rumelhart, 1980, 1984; Anderson, 1984).

The camera metaphor was also used in the late 1970s to bring a second problem to the attention of the proponents of saved prior knowledge, although the problem was directly aimed at deep-structure story grammarians: prior knowledge structures were shown to resist change and, as a result, they were more likely to be doubly in the way of understanding than fostering it (Iran-Nejad, 1980/1987). This was illustrated using a surprise-ending story by Thurmond (1978). The point made was that deep-structure templates were static long-term memory patterns. Consequently, they were stable to the point of allowing no change at all. This was so especially in their embodied long-term memory form, in which static embodied forms were as unchanging as statues (Miller, 1978)—they were permanently inordinately stable. To be sure, surprise-ending stories like Thurmond’s also needed the benefit of inordinate stability; but to tolerate radical change, they had to be, paradoxically, inordinately changeable at the same time. Biofunctional systems allow the simultaneous capability of inordinate stability and unrestrained flexibility because they can readily create knowledge on demand. Miller (1978) illustrated how dynamic systems do this using the analogy of the shape of a fountain and contrasted it with the change-resistance capacity of static structures like the statue (Iran-Nejad et al., 1981/1984).

The Thurmond (1978) story, for example, was about a nurse, Marilyn. One late night in a large city, she leaves work at a hospital, where she had recently attended to patients badly beaten by a mugger in the area. Driving home on the freeway, she notices that she is running out of gas and debates what to do. Thinking about the mugger in the area and scared, she exits the freeway heading toward the station where she knows the friendly attendant, Gabriel. He fills, cleans the car windows, and when she is about to leave, he insists that she goes inside the station office first to see a birthday gift from his sister. She parks as he signals and follows him inside. Once there, he turns around, locks the door, and pulls a gun out of the drawer. Too frightened to defend herself, she begins experiencing the symptoms of shock as she watches him staring haggardly outside the window with lips moving. Finally, she hears him saying “Sorry, I had to scare you like that. I did not know what else to do when I saw that dude hiding on the floor in the back of your car. I will call the cops now.”

In this relatively organically sophisticated storyline, up until the moment of surprise, readers entertain an inordinately stable understanding. In this pre-surprise understanding, the friendly Gabriel is seen as a wolf in sheep’s clothing. Then, at the moment of surprise there is a dramatic, rapid-strike flip-flop in understanding resulting in readers seeing Gabriel as a Good Samaritan. Remarkably, the storyline causes two mutually incompatible perspectives on one and the same exact text of the story, one way of understanding immediately after another with less than 2 s in between. Administering the rapid-strike flip-flop takes dismissing one understanding and re-assigning another to the relatively long text of the same story. This happens spontaneously without having to go back to actively recall and re-allocate attention to every word, phrase, and sentence of the text all over again (Schallert, 1982; Iran-Nejad, 1986, 1989a,b,c). If one were to assume tightly knit deep structures for every phrase and sentence in the story (see Rumelhart, 1975), instantaneous reorganizations like the one in the Thurmond story would be difficult to imagine, let alone to explain and actively enact.

How did the manual camera play the metaphoric role assigned to it out of desperation in the late 1970 to shed light on this storyline? Clearly, as demonstrated then in the form of a challenge to deep-structure story grammarians, adding the long-winded prior knowledge vernacular could do very little than being in the way. Without the prior knowledge vernacular, the metaphoric role of the manual camera was rather straightforward. In the absence of pre-existing frames, it would simply take a well-built manual camera in the hands of a life-long photographer with flawless professional artistry to rapid-snap in immediate consecution two pictures of a single scene from two different angles. This would not make a perfectly tight metaphor for replicating the manner and nature of biofunctional embodiment of understanding but would be close enough of an approximation to show that no saved prior knowledge would be necessary and any would be in the way.

### Knower Control Processes^1^

It takes two corequisite sets of control processes to explain the manner of the biofunctional embodiment of understanding without resorting to any saved prior knowledge and do so over and beyond what was said above in the context of the manual camera metaphor (Iran-Nejad, 1990; Iran-Nejad and Chissom, 1992; Iran-Nejad et al., 1992), including the rapid-strike feats of multiple-source understanding (Iran-Nejad, 1986, 1989c) and enjoying (Diener and Iran-Nejad, 1986; Iran-Nejad, 1987; Iran-Nejad and Cecil, 1992) the likes of the Thurmond (1978) surprise-ending story (Iran-Nejad, 1983a,b). The first set, prerequisite for the second, includes processes like realization, recognition, revelation, hearing, seeing, appreciating, grasping, getting, understanding, clicking, apprehending, insight, and the like. Members of this set are knowthat, as opposed to knowhow, processes (Bransford and Schwartz, 1999); they rise spontaneously as a function of the immediate ground of multiple-source biofunctional understanding by the key process of biofunctional knowing by revelation, as opposed to by recall; they are called simply biofunctional understanding (BU) processes because they are caused by the immediate flow of ongoing biofunctional activity; and they present themselves to the unwary knower, unbeckoned and in an after-the-fact manner, all with the extraordinary but characteristic click of unmistakably understanding, albeit, at varying degrees of strikingness or surprise (Iran-Nejad, 2000; Prawat, 2000). These processes assume no saved prior or any other kind of knowledge; it is to these processes that, in part, the immediate analogy of the manual camera applies; and it is these processes that are the pure wisdom operators of the physical intellectual capacity of biofunctional understanding. The second set, post-requisite to the first, includes thinking, concentration, contemplation, meditation, prediction, foresight, hindsight, elaboration, application, evaluation, observing, listening, looking, and so forth. These processes are the source and operators of conceptual understanding (CU); they represent the key process of understanding fresh realizations further by reflection; they make up the wisdom of the intellectual capacity of knowing on demand; and they are dependent for their operation on the “active I” process—the third and only other source of contribution to the Iran-Nejad wholetheme spiral of biofunctional understanding and critical thinking (Iran-Nejad, 1978, 2000; Iran-Nejad and Gregg, 2001; Iran-Nejad and Irannejad, 2017a,b). Finally, it is this latter set of processes that links the embodied mind and biofunctional theories supportively and turns into oxymorons the theories of embodied cognition and biofunctional understanding. The two sets of understanding processes function differently, albeit complementarily, in the spiral of biofunctional understanding (Iran-Nejad and Irannejad, 2017b).

The BU and CU processes differ in the manner they relate to spontaneous systemic control relative to the third process—the active I” or the person of the knower. BU processes sharpen the ground for cohesion sensing (e.g., spontaneous curiosity) and CU processes serve the cause of cohesion seeking (e.g., active questioning) on the part of the agent or the person of the knower in an overall physical system of diverse subsystems and microsystems (Caligiore et al., 2017). More specifically, CU processes like thinking and reflection may be described as cohesion seeking attention-allocation processes; BU processes such as realization and grasping may be described as cohesion-sensing. Substantial direct and indirect evidence suggests that embodied systems may contribute to understanding as long as the knower uses available “knowthat” content to keep the cohesion-seeking cursor of attention-allocation going on embodied systems as a necessary but insufficient condition for the CU set of control processes to play their role (Iran-Nejad and Irannejad, 2017b). The other necessary condition for CU processes is to serve as the corequisite knowhow for enabling systematic cohesion-seeking on the part of the knower. Accordingly, cohesion-sensing and cohesion-seeking make up the necessary and sufficient conditions for the person of the knower to stay actively involved in attention-allocation to embodied systems thereby combining the contributions of the available (a) declarative content and (b) procedural content. In short, CU is something the knower must (a) knowthat the knower does to keep the cursor of attention-allocation going on fresh revelations caused by embodied systems and (b) the knower must also knowhow to do the same; and do so systematically.

Thus, BU and CU control processes work corequisitely. The BU processes have to do with the contributions of the spontaneous cohesion sensing ground of the intellectual capacity of biofunctional understanding. This is the spontaneous wisdom of the cohesion sensing intellectual capacity of the physical biology. By contrast, the CU processes represent the deliberate wisdom of the cohesion-seeking intellectual capacity of metaphysical knowing. Knowers may deliberately allocate attention to the clicks of understanding coming in the form of, e.g., realizations. They may do so by means of the knowthat revelation content delivered in those realizations, using the knowthat content to allocate attention to the ongoing flow of cohesion, doing whatever it is supposed to be spontaneously doing, e.g., causing clicks of BU. However, the knower must also know (e.g., in order to avoid wild-goose chases after non-existent, unnecessary, irrelevant, or even superstitious knowhows), paradoxically, that the knower does not have to have the knowhow. Here, understanding as cohesion-sensing must come from the corequisite relation between two types of knowledge. In this case, in some very fundamental way, cohesion-seeking works with cohesion-sensing biofunctional understanding in a manner like “fishing” sense or meaning out of ongoing biofunctional activity without, paradoxically, even knowing how to fish but waiting patiently for the fish to surprise by jumping into one’s lap.

### The Old and the New in the Long Story Made Short

The long story made short so far in this introduction (Iran-Nejad, 1978, 2000; Prawat, 2000; Johnson, 2015) tells something radically, if not paradoxically, new about something intuitively, if not otherwise, old. Biology is the spontaneous systemic source of the wisdom we have always known and called understanding. As a whole, this idea is, in part, radically new because it lifts dramatically in our minds the biofunctionally alive, well, and still running biological system from the status of the vegetative organ it has always unfairly, if not unethically, held to the new status of the wisdom source it is expectedly going to hold from here on. As a whole, the idea is, in part, old because it now holds inside something we have always known, namely, the intellectual wisdom capacity we call understanding.

For the sake of experimental biofunctional science, and while we are on the topic of something new and something old, the next BU and CU examples assume that the biofunctional process of understanding works analogously to the biofunctional process of salivation, housing some of the oldest and widely recognized and used variables in the experimental-science paradigm of Pavlovian classical conditioning. Conceptually, as in both CU and BU, we knowthat we salivate but, unlike in CU, we also knowthat, paradoxically, the biofunctional process of salivation is not something within the reasonable realm of our conceptual knowhow. We know that we get a steady stream of saliva in our mouths because we sense its post-production presence (i.e., its effect) in there; but we also know contently that getting saliva in our mouth does not have to be within the realm of our conceptual doing. Unlike for CU, we are simply content and thankful, so to speak, that it gets there; and we are untroubled by the absence in our conceptual understanding of the “how” of the biofunctional process that happens to make sure that it is there as needed to play its vital corequisite role. The fundamental working assumption of the biofunctional theory is that biofunctional understanding occurs in an analogous manner. Knowers know that they understand because they sense the steady stream of its post-production understanding clicks at varying degrees of strikingness; and their sense of conceptual curiosity is ordinarily as unimpressed with the absence of the “how” of biofunctional understanding as it is by the how of biofunctional saliva production. If so, we predict that our study participants should, at least in principle, tend to agree intuitively, e.g., not only with the statement *I know that I salivate* [biofunctionally] *even though I myself do not really know* [conceptually] *how to salivate* but also analogously with the statement *I know that I understand people* [biofunctionally] *even though I do not really know* [conceptually] *how to understand people.*

## Materials and Methods^2^

### Purpose and Rationale

The present study tests the *a priori* prediction, derived from the Iran-Nejad wholetheme spiral of biofunctional-understanding, that CU and BU processes appeal differently in cohesion sensing to knower control; and that CU and BU statements may be used, with ample caution, to carry out the test. More specifically, the CU statements contain both knowthat and knowhow content as corequisites. By contrast, BU statements carry knowthat content but the knowhow content is conspicuously absent in them. Since the two types of statements (a) are identical in format, (b) the format employed pits the declarative knowthat and the procedural knowhow types of content against each other, (c) both statements carry knowthat contents in them, and (d) the knowhow content is absent only in the BU statements, therefore, CU statements are expected to be rated in cohesion sensing as less coherent than BU statements. We report two experiments next. Experiment 1 tests our *a priori* prediction and Experiment 2 is expected to replicate the results of the first experiment.

### Design

We employed a one-way design with two levels of Statement as a within-subjects factor. In two experiments, participants read statements like the following for internal consistency, as response time was recorded. The two types of statements are otherwise identical in format and other but not all respects; and they should be relatively well-suited for this early-stage investigation.

(1) CU1 *I know that I think about the topics I consider even though I myself do not really know how to think about the topics I consider* (relatively more incoherent).
(2) BU1 *I know that I understand the topics I consider even though I myself do not really know how to understand the topics I consider* (relatively less incoherent).
(3) CU2 *I know that I swallow my saliva from time to time even though I myself do not really know how to swallow my saliva from time to time* (analogy: not used in the study).
(4) BU2 *I know that I get a steady stream of saliva in my mouth even though I myself do not really know how to get a steady stream of saliva in my mouth* (analogy: not used in the study).

We used a survey comprising 22 BU and 22 CU statements. Cronbach’s Alpha was 0.88 for BU statements and 0.90 for CU statements. An additional CU example with both knowthat and knowhow content present in it was CU3 *I know that I pay more attention to main ideas even though I myself do not really know how to pay more attention to main ideas.* Therefore, this negative statement was expected to represent a false first-person claim and be rated on the relatively lower end of the internal consistency scale, compared to BU statements. For this CU statement, the claim of phenomenological certainty in knowing that one pays more attention to main ideas must carry corresponding phenomenological certainty about the corequisite knowing how to pay more attention to main ideas. As a result, negating the knowhow is expected to conflict with the assertion of the knowthat and cause inconsistency. Contrariwise, the following BU example is expected to represent a true first-person claim and be rated as relatively more internally consistent, compared to CU statements: BU3 *I know that I experience clicks of understanding inside me every now and then even though I do not really know myself how to experience clicks of understanding inside me every now and then.* This (true) statement was predicted to be rated as more consistent than CU statements in internal consistency. Knowing that one experiences clicks of understanding has no corequisite knowhow for experiencing those clicks because those clicks are the work of biofunctional understanding

### Participants

A total number of 34 students from the same graduate Educational Psychology course in the College of Education (21 women, 13 men; *M* age = 25, *SD* = 3.4) participated one semester apart in two studies (N Fall semester: 17, N Spring semester = 17) in exchange for course credit. All students who were contacted volunteered to participate and completed the survey with no missing values. A power analysis, using the GPower software package (Faul and Erdfelder, 1992), revealed that the sample size of 17 was sufficient. The recommended effect sizes ranged between small (f 2 = 0.02), medium (f 2 = 0.15), and large (f 2 = 0.35) (see Cohen, 1977) and the alpha level was *P* < 0.05. The analysis showed that the statistical power was 1.00 which exceeded 0.99 for the detection of strong (perfect) power at the large effect size level (0.671). The purpose of the second experiment was to replicate the first.

### Procedure

The two sets of statements were presented to participants in Qualtrics version 2013 available online at www.qualtrics.com. The order of presentation was fully randomized. The instructions informed the participants to rate the internal consistency of each statement on a five-point Likert scale ranging from 1 (not consistent at all), 2 (somehow consistent), 3 (consistent), 4 (very consistent), to 5 (extremely consistent). Participants received the Qualtrics link to the study by e-mail. Clicking on the link took the participants first to an IRB-approved informed consent form followed by brief instructions with an example of each type of statement. The participants rated the statements as the program recorded the rating response time between key presses.

### Data Analysis

For the first analysis, two mean consistency rating (CR) scores were calculated over the 22 items within each statement type to obtain two mean scores, one for CU and one for BU statements for each participant. Similarly, two mean character response time (CRT) scores were calculated. First, for each participant, the time in seconds to rate the consistency of each statement was divided by the number of characters and spaces in that statement to obtain a response time in seconds per character. Then, a mean CRT was calculated across the 22 BU and the 22 CU items. Averaging across statements was deemed reasonable because these items were presented to each participant in a fully randomized order. The four means thus obtained for each participant were used as dependent measures in subsequent analyses. For each of the two studies reported, two one-way repeated-measures ANOVAs were used, one for each of the two dependent variables, with two levels of statement type (BU, CU) as a within-subjects variable. Subsequently, a set of linear mixed model (LMM) analyses were also conducted for both studies to confirm the findings of the first analyses. According to McCulloch and Searle (2000) for analyses such as repeated measures of survey respondents, it is common for the data to be correlated and thus, mixed models are used to extend the repeated measure models in GLM. In the present study, correlated data were possible, even though less-likely given that statement items were presented to each subject in a fully randomized order. Therefore, reporting the LMM results was deemed appropriate.

## Results

### Experiment 1

**FIGURE 1:**
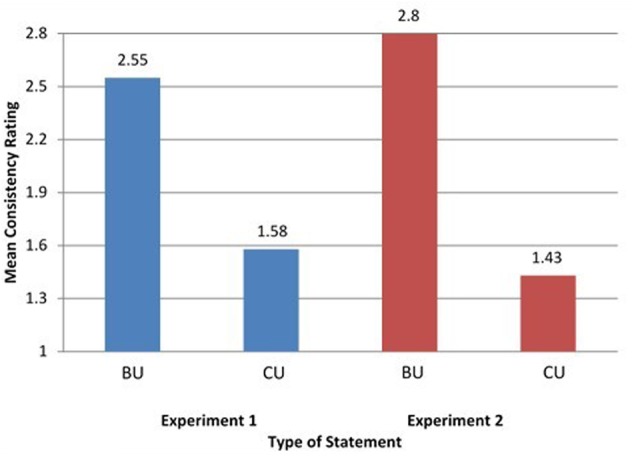
Mean consistency rating for biofunctional understanding (BU) and conceptual understanding (CU) statement types for Experiments 1 (blue) and 2 (red).

**FIGURE 2:**
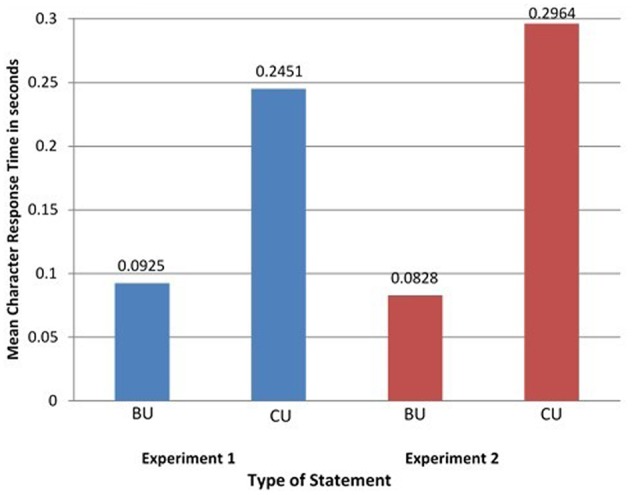
Mean character response time (in seconds) for biofunctional understanding (BU) and conceptual understanding (CU) statement types for Experiments 1 (blue) and 2 (red).

As predicted, participants rated CU statements significantly less internally consistent than BU statements (see **Figure 1**, left panel), *F*(1,16) = 25.643, *P* < 0.001, η^2^= 0.616 (*M_BU_* = 2.55, *SD* = 0.65; *M_CU_* = 1.58, *SD* = 0.66). Similarly, the results of the analyses of the response time revealed that participants responded significantly more slowly to CU than BU statements (see **Figure 2**, left panel), *F*(1,16) = 7.53, *P* < 0.014, η^2^= 0.32 (*M_CRT/BU_* = 0.0925, *SD* = 0.045; *M_CRT/CU_* = 0.2451, *SD* = 0.22). Thus, the findings confirmed the *a priori* predictions of the study about the presence/absence of corequisite content knowledge differences in rated internal consistency and response time between CU and BU statements. Follow-up LMM analyses with fixed levels of statement type (CU, BU) and levels of statement items (44) set to be random confirmed the results. For consistency ratings, there was a significant effect for statement type, *F*(1,725) = 47.319, *P* < 0.000. This effect was also significant for response time, *F*(1,725) = 12.083, *P* < 0.001.

### Experiment 2

The results of Experiment 1 supported the hypothesis that the knower control processes for CU such as thinking and contemplation are different from those for BU like realization and revelation. Experiment 2 used the exact same methodology as Experiment 1 for the purpose of replication. Data analysis of the consistency rating (CR) scores confirmed the results of the first study. Subjects rated the BU statements significantly more internally consistent than CU statements (**Figure 1**, right panel), *F*(1,16) = 29.65, *P* < 0.001, η^2^= 0.679, *M_BU_* = 2.80, *SD* = 0.85, *M_CU_* = 1.43, *SD* = 0.53. The results of the analyses of the CRT scores revealed that there was also a significant difference between participant responses to BU and CU statements (**Figure 2**, right panel), *F*(1,16) = 8.626, *P* < 0.001, η^2^= 0.381, *M_CRT/BU_* = 0.0828, *SD* = 0.05188, *M_CRT/CU_* = 0.2964, *SD* = 0.2717. Follow-up LMM analyses also confirmed the results both for consistency ratings, *F*(1,725) = 18.012, *P* < 0.001 and response time, *F*(1,725) = 11.208, *P* < 0.001.

Comparison of the left and right panels in the two Figures shows that the results of Experiment 2 closely matched those of Experiment 1. The two adjacent panels in each figure reveal the same pattern of results for the two experiments. As already said, all statements used the same exact format and normal semantic content; and they all contained knowthat knowledge. They differed, however, in the degree they did or did not carry knowhow content. In both studies, as expected, CU statements (that contained knowhow content) were rated as being more internally inconsistent and showed slower response time relative to BU statements that were characterizable by the relative absence of conceptual how content.

## Discussion, Conclusion, and Future Directions

### Knowledge Everywhere, and Not a Faint Sign of Understanding Anywhere

Historically, knowing and understanding have been regarded as one and the same intellectual capacity. As a result, studies of understanding have been non-existent in the midst of widespread investigations of knowledge. The default assumption has been that today’s accumulation of basic scientific research on knowing, aided by physical science’s brand of systematic observation, is allowing us for the time being to separate the relevant grains of fact from the irrelevant chaff of fiction in the realm of knowing and will, in all likelihood, some day serve the cause of tomorrow’s understanding. The most characteristic attributes of this attitude in favor of knowledge at the expense of understanding have been inordinate stability of thinking about knowledge and resistance to change in favor of understanding. To add one more example to the literature cited in the introduction, Piaget’s developmental research has been all about knowledge and none about understanding. For another different example, Grimm (2006) acknowledged that every serious *epistemologist* has denied the interchangeable relationship between knowing and understanding, but then Grimm himself went on to make the case again for the seductive idea that understanding is a species of knowledge.

The present study sought evidence for the opposite viewpoint—that knowing and understanding are different, in fact mutually corequisite, and complementary intellectual capacities; and they embody contrastively in their relative causes and consequences. The goal of this article, as described in the introduction, was to present the first original research study of the two main sets of dynamic and active control processes that integrate in relative cohesion the intellectual capacities of knowing and understanding into a wholetheme spiral of biofunctional understanding. Backed by evidence of the kind obtained in the original research reported in this article, the spiral promises to shed light on the historically dark ground of uncertainty, both in theory and method, surrounding the manner and nature of understanding. Given the transparent outline of the spiral in the introduction as the intrinsic context for the two sets of control processes targeted in the study, the evidence from the experiments reported—the first of its kind from where the non-existent state of the art in the experimental science of understanding stands at this early stage in the development of embodiment science—supported the *a priori* predictions tested in one experiment and replicated in a second experiment.

### A Different Kind of Consideration

Perhaps the non-existent state of the art of the experimental science of biofunctional understanding (ESBU) is ominously symptomatic of something too different altogether to expect from our existing state of the art in the experimental science of knowing that is confined today to the “prison house” of conceptual understanding (ESCU, see, e.g., Prawat, 2000; Iran-Nejad and Irannejad, 2017a). In other words, the sacred run of the mill in ESCU is something to which we have grown too accustomed as a comfort zone, which makes it something too frustrating to question. Nevertheless, question we must before we may figure out that hitherto-inconceivable way that must give us the new pair of feet for walking across the no man’s land that Eleanor Rosch identified between today’s ESCU and tomorrow’s ESBU. Intriguingly, as we have been trying to show in this article, it is too simplistic to blame the bloom in ESCU for the doom in ESBU; nor is it realistic to wish for the bloom of ESBU to flourish in the doom of ESCU. The two-horn beast of the challenge we are facing is analogous to presenting the future scientists with the challenge of having their cake and eating it as well. Remarkably, that is exactly what the wholetheme spiral of biofunctional understanding promises us to be able to do. If so the findings of the two experiments reported here may represent, their limitations in the lights of both ESCU and ESBU notwithstanding, a distant ray of light at the end of the dark tunnel of the history of the intellectual capacity of understanding. Therefore, before we take another step toward experimenting with understanding, we must first take a good look at today’s experimental science paradigm. In fact, this was the assumption with which the present investigation began and, now having completed the study for it, it might not be too farfetched a conclusion to palate.

### Physical Science’s Guiding Principle of Systematic Observation

The main problem with today’s experimental science is its exclusive reliance for a guiding principle on the physical science’s systematic observation. With this principle in hand, we join Shulman (1999) to reminisce fondly with the comforting simplicity of behavioral science and sadly with the dismayingly unmanageable and possibly pseudo complexity of cognitivism, as behavioral scientists had predicted and cognitivists miscalculated. Of course, luckily today’s cognitive science is an interdisciplinary science, which could include the embodied science of biofunctional understanding. The real problem with the experimental science of today is the extent of its overreliance on its one and only guiding principle of sensory observation based on two seductive assumptions, both of which can be readily shown to be flawed. One assumption is that sensory observation offers the most immediate window to the so-called physically observable world. The second assumption is that the sensory modalities have the widest and the most immediate contact with the real world. In fact, it is possible to show that it is the biofunctional modality that has the widest and the most immediate contact with the real world, which includes contact through the senses as well.

### Systematic Observation and Analytic Fact-Seeking

It may appear otherwise, but it may be fair to say, as suggested by Shulman, that behavioral scientists successfully transitioned psychology to science at the expense, fairly or not, of conceptual understanding and other unobservable mental states as overly subjective threats to the objective science of the kind established according to the powerful physical-science’s guiding principle of systematic observation. Later, cognitive psychologists adopted the principle of hard and observable external objects and, encouraged by the soft and immediately unobservable nature of the computer program analogy (Neisser, 1967; Iran-Nejad and Winsler, 2000), generalized it to the soft and unobservable internal representations of the hard and observable external objects. According to the guiding principle of systematic observation, the hard external-world and its soft internal representation are, inherently or through the subjective eyes of study participant beholders, shrouded in unsystematic complexity. The goal of analytic science of cognition was to simplify complexity of mental representations by isolating observable facts in the form of declarative propositions, e.g., *Mindfulness enhances critical thinking* (Noone et al., 2016). These propositions could, then, be framed into binary if-question hypothesis testing, aimed at separating the significant gold of true propositions, discarding the insignificant chaff of false propositions, add the new truths in the form of basic scientific knowledge to the previously stored wealth of basic or pragmatically useful knowledge in external-media (e.g., textbooks) or internal long-term memory stores (e.g., hierarchical semantic networks). Subsequently, these soft but storable scientific (i.e., systematically-derived) facts might be uploaded or downloaded for the purposes of replication, generalization, and application. If we assume that knowledge and understanding are one and the same intellectual species, this is the tragic end of the story for human understanding. In the realm of the fact seeking field of cognition, the difference between the hard and the objective and the soft and the subjective is stark but confounded, making conceptual understanding abstract, subjective, and a bemuddling scientific liability.

For many decades, interested investigators have puzzled over the challenges that understanding-related factors such as motivation and transfer present to the community of experimental cognitive researchers. The reasons behind the challenging state of the art have been diverse; but they are all traceable to the study of cognition or knowledge in isolation. Among these investigators are leading practitioners like Bransford et al. (1977, 2000), Schön (1983), Bloom (1984), Shulman (1986, 1999), McCombs (1991), Gardner and Boix-Mansilla (1994), Willis (2000), and Salomon (2006). These scholars of science and practice have keenly observed the problem and its dismaying consequences in the trenches of the real world of practice. Shulman (1999), for example, pointed out.

After I finished graduate school and first began teaching the psychology of learning, I was confident that I really understood what the process of learning entailed. However, over the past 35 years, I have systematically studied learning and understanding in many contexts, and I have taught many courses on the subject. Alas, my understanding has now become more complex, vague, and somewhat ambiguous.

Having voiced concerns like this, Shulman spoke of the consequences as pathologies of which he named three: “we forget, we don’t understand that we misunderstand, and we are unable to use what we learned. I have dubbed these conditions *amnesia, fantasia, and inertia*” (italics in original).

If we assume that factual knowledge and biofunctional understanding are different, we enter the new realm of the hard and unobservable biological systems and must deal with the evidence of the kind reported in the present study in this new light.

### Biofunctional Science’s Guiding Principle of Systematic Consensus in Systemic Cohesion

The present study made use of participant subjective reports, a methodological liability if viewed solely through the objective fact-seeking lens of analytic cognition. As reviewed in the introduction, a growing literature now embraces the theory that the physical biology is a diverse—color-coded, so to speak—source of special systemic functions (Iran-Nejad, 1980/1987). Among these are the special systemic sources that support the embodied-mind functions (Iran-Nejad and Ortony, 1984; Iran-Nejad and Gregg, 2011; Borghi et al., 2013; Alverson, 2015; Jin et al., 2015, 2016; Scorolli and Borghi, 2015; Caligiore et al., 2016; Thill and Twomey, 2016). Chief among these functions are those having to do with the newly discovered idea that physical biology is the direct and immediate source of the hitherto-neglected wisdom of the intellectual capacity of biofunctional understanding that is the principle contributor to the systemic spiral of biofunctional understanding. Therefore, it is possible to show how human understanding is, by virtue of its fundamentally consensual nature, uniquely characterizable by systemic cohesion sensing, cohesion seeking, and, thereby, systematic science-quality consensus-seeking.

Given this line of reasoning, the newly found direct and immediate wisdom of biofunctional understanding frees embodiment science from the confining prison house of systematic fact-seeking observation (Iran-Nejad and Irannejad, 2017a). In this light, subjective data-gathering of the type done in the present study is a methodological asset rather than a subjective liability. Specifically, the spiral of biofunctional understanding spontaneously delivers its extraordinary clicks of understanding in systemic cohesion with affectively rich revelations (Iran-Nejad, 1987). Subsequently, the “active I” may use the knowthat results of the spontaneous revelations by immediate means of direct systemic cohesion sensing and seeking to engage in further conceptual understanding by reflection (Iran-Nejad et al., 2015). Therefore, in the embodied flow of the revelation-producing spiral of biofunctional understanding, subjective sense-making and sense-reporting find a new, indispensable, and unique methodological role to play (Iran-Nejad and Irannejad, 2017b, p. 3). Thus, in the science of biofunctional embodiment, the physical science’s principle of systematic observation is a necessary but insufficient front for science making. What is needed, in addition, is the complementary guiding principle of systematic sensemaking backed by systematic consensus making. It gets a bit long-winded here to say given the available space, but it is in the light of the unified function of this immediate and direct (a) systemic cohesion sensemaking, (b) systematic consensual sensemaking, with (c) potential backing from systematic science-quality consensus-making that the methodology and the findings of this study must be evaluated.

The finding of the difference between CU and BU supports the idea that the paradox of the missing “how” of the (physical) biofunctional understanding is real and within the grasp of systemic cohesion sensing of study participants and systematic consensus-seeking among professional scientists. There are indications that exemplary scientists like Einstein and Pasteur make systematic use of this spontaneous capacity for cohesion in their science (Iran-Nejad, 2016). At the level of study participants, compared to thinking, college students in the present experiments seemed to be content not knowing how to understand even though they knew, paradoxically, that they did understand. An intriguing implication is that knowers at all levels from naïve study participants to advanced scientists may (be encouraged to) engage in systemic body-mind cohesion-sensing as well as consensus-seeking (Caligiore et al., 2016). Further supportive evidence has been reported in a semester-long classroom intervention study in which undergraduate teacher education students were encouraged to seek their own first-person revelations and engage in writing to reflect on them (Iran-Nejad et al., 2015). Therefore, there is hope for new embodiment-science methodology (Caligiore et al., 2016; Iran-Nejad and Irannejad, 2017a,b), that the physically hard and forbidding black box of the physical body may have now developed access windows for airing its infinite wisdom and for the light of systemic cohesion-sensing to shine through as directed by science-quality sources of systematic consensus.

## Ethics Statement

This study was carried out in accordance with the recommendations of The University of Alabama Institutional Review Board. All subjects gave written informed consent in accordance with the Declaration of Helsinki. The protocol was approved by The University of Alabama Institutional Review Board (IRB # 12-OR-392-R1).

## Author Contributions

AI wrote the article. FB programed and ran experiments, helped with the method and results sections including experimental material and data analysis, and read and commented on drafts.

## Conflict of Interest Statement

The authors declare that the research was conducted in the absence of any commercial or financial relationships that could be construed as a potential conflict of interest.

## Footnotes

1 The theme of this article hinges around the role of the intellectual capacity of knowing in conceptual control. Accordingly, it is deemed advisable for the sake of wholetheme cohesion to use derivatives like knower over the more ambiguous learner, knowhow over the less concise “knowing how,” and knowthat over the less uniform and, for the time being, more standard “knowing that.” Therefore, knower is used here interchangeably with the “active I,” knowthat with declarative content, and knowhow with procedural content.

2 It should be clear by now that knowthat, knowhow, and the like are special-status variables in content knowledge. It is also clear that time and again investigators have turned and returned to these variables; but treatments have seldom reached beyond content *per se* (Ryle, 1949; Williams, 2008). In relatively recent years, educational researchers have employed the factor of active control in learning (Brown, 1975; Bransford and Schwartz, 1999) and conceptual understanding (Shulman, 2002) as used in the present article (Iran-Nejad, 1990; Iran-Nejad and Chissom, 1992). It is in the background of this research and the literature we have reviewed in the introduction that we are presenting here what is, to our knowledge, the first experimental study of the relationship between knowing and understanding.

## References

[B1] Adams F. (2010). Embodied cognition. *Phenomenol. Cogn. Sci.* 9 619–628. 10.1007/s11097-010-9175-x

[B2] Alba J. W. Hasher L. (1983). Is memory schematic? *Psychol. Bull*. 93 203–231. 10.1037/0033-2909.93.2.203

[B3] Alverson R. (2015). A biofunctional perspective on learning environments. *Front. Psychol.* 6:1973. 10.3389/fpsyg.2015.01973 26779048 PMC4688337

[B4] Anderson R. C. (1977). “The notion of schemata and the educational enterprise: General discussion of the conference,” in *Schooling and the Acquisition of Knowledge* eds Anderson R. C. Spiro R. J. Montague W. E. (Hillsdale, NJ: Erlbaum) 415–431.

[B5] Anderson R. C. (1984). Some reflections on the acquisition of knowledge. *Educ. Res.* 13 5–10. 10.3102/0013189X013009005

[B6] Anderson R. C. Spiro R. J. Anderson M. C. (1978). Schemata as scaffolding for the representation of information in connected discourse. *Am. Educ. Res. J.* 15 433–440. 10.3102/00028312015003433

[B7] Atkinson R. C. Shiffrin R. N. (1968). “Human memory: a proposed system and its control processes,” in *The Psychology of Learning and Motivation* Vol. 2 eds Spence K. Spence J. (New York, NY: Academic Press) 89–195.

[B8] Beers T. (1987). Schema-theoretic models of reading: humanizing the machine. *Read. Res. Q.* 22 369–377. 10.2307/747974

[B9] Billing E. A. Svensson H. Lowe R. Ziemke T. (2016). Finding your way from the bed to the kitchen: reenacting and recombining sensorimotor episodes learned from human demonstration. *Front. Psychol.* 3:9 10.3389/frobt.2016.00009

[B10] Black J. B. Wilensky R. (1979). An evaluation of story grammars. *Cogn. Sci.* 3 213–230. 10.1044/0161-1461(2011/10-0038) 21969531 PMC3387811

[B11] Bloom B. S. (1984). The 2 sigma problem: the search for methods of group instruction as effective as one-to-one tutoring. *Educ. Res.* 13 4–16. 10.2307/1175554

[B12] Borghi A. M. Scorolli C. Caligiore D. Baldassarre G. Tummolini L. (2013). The embodied mind extended: using words as social tools. *Front. Psychol.* 4:214. 10.3389/fpsyg.2013.00214 23641224 PMC3640182

[B13] Bransford J. D. Brown A. L. Cocking R. R. (eds) (2000). *How People Learn: Brain, Mind, Experience, and School.* Washington, DC: National Academy Press.

[B14] Bransford J. D. Johnson M. K. (1972). Contextual prerequisites for understanding: some investigations of comprehension and recall. *J. Verbal Learning Verbal Behav.* 11 717–726. 10.1016/S0022-5371(72)80006-9

[B15] Bransford J. D. McCarrell N. S. Franks J. J. Nitsch K. E. (1977). “Toward un explaining memory” in *Perceiving Acting, and Knowing: Toward an Ecological Psychology* eds Shaw R. E. Bransford J. D. (Hillsdale, NJ: Erlbaum) 31–55.

[B16] Bransford J. D. Schwartz D. L. (1999). “Rethinking transfer: a simple proposal with multiple implications,” in *Review of Research in Education* Vol. 24 eds Iran-Nejad A. Pearson P. D. (Washington, DC: American Educational Research Association) 1–19.

[B17] Brewer W. F. (2000). Bartlett, functionalism, and modern schema theories. *J. Mind Behav.* 21 5–35.

[B18] Brewer W. F. Lichtenstein E. H. (1981). “Event schemas, story schemas, and story grammars,” in *Attention and Performance* Vol. 9 eds Long J. D. Baddeley A. D. (Hillsdale, NJ: Erlbaum) 363–379.

[B19] Brewer W. F. Nakamura G. V. (1984). “The nature and functions of schemas,” in *Handbook of Social Cognition* Vol. 1 eds Wyer R. S. Srull T. K. (Hillsdale, NJ: Lawrence Erlbaum) 119–160.

[B20] Brown A. L. (1975). “The development of memory: knowing, knowing about knowing, and knowing how to know,” in *Advances in Child Development and Behavior* Vol. 10 ed. Reese H. W. (New York, NY: Academic Press) 103–152. 10.1016/s0065-2407(08)60009-9 1101659

[B21] Caligiore D. Pezzulo G. Baldassarre G. Bostan A. C. Strick P. L. Doya K. (2016). Consensus paper: towards a systems-level view of cerebellar function: the interplay between cerebellum, basal ganglia, and cortex. *Cerebellum* 16 203–229. 10.1007/s12311-016-0763-3 26873754 PMC5243918

[B22] Caligiore D. Pezzulo G. Baldassarre G. Bostan A. C. Strick P. L. Doya K. (2017). Consensus paper: towards a systems-level view of cerebellar function: the interplay between cerebellum, basal ganglia, and cortex. *Cerebellum* 16 203–229. 10.1007/s12311-016-0763-3 26873754 PMC5243918

[B23] Clark A. (2008). *Supersizing the Mind: Embodiment, Action, and Cognitive Extension.* New York, NY: Oxford University Press 10.1093/acprof:oso/9780195333213.001.0001

[B24] Cohen J. (1977). *Statistical Power Analysis for the Behavioral Sciences.* Mahwash, NJ: Erlbaum.

[B25] Collins A. M. Loftus E. F. (1975). A spreading activation theory of semantic processing. *Psychol. Rev.* 82 407–428. 10.1037/0033-295X.82.6.407

[B26] Craik F. I. M. Lockhart R. S. (1972). Levels of processing: a framework for memory research. *J. Verbal Learning Verbal Behav.* 11 671–684. 10.1016/S0022-5371(72)80001-X

[B27] Craik F. I. M. Tulving E. (1972). Depth of processing and the retention of words in episodic memory. *J. Exp. Psychol. Gen.* 104 268–294. 10.1037/0096-3445.104.3.268 24156261

[B28] Diener E. Iran-Nejad A. (1986). The relationship in experience between various types of affect. *J. Pers. Soc. Psychol.* 50 1031–1038. 10.1037//0022-3514.50.5.1031

[B29] Edelman G. M. (2006). *Second Nature: Brain Science and Human Knowledge.* New Haven: Yale University Press.

[B30] Faul F. Erdfelder E. (1992). *GPOWER: A Priori, Post-Hoc, and Compromise Power Analyses for MS-DOS.* Bonn: Bonn University Department of Psychology.

[B31] Gardner H. Boix-Mansilla V. (1994). Teaching for understanding in the disciplines—and beyond. *Teach. Coll. Rec.* 96 198–218.

[B32] Gärtner C. (2011). Wisdom in the flesh: embodied social practices of wisdom in organisations. *Phil. Manag.* 10 29–42. 10.5840/pom20111019

[B33] Gärtner C. (2013). Cognition, knowing and learning in the flesh: six views on embodied knowing in organization studies. *Scand. J. Manag.* 29 338–352. 10.1016/j.scaman.2013.07.005

[B34] Ghorbani N. Watson P. J. Farhad M. Chen Z. (2014). A multi-process model of self-regulation: influences of mindfulness, integrative self-knowledge, and self-control in Iran. *Int. J. Psychol.* 49 115–122. 10.1002/ijop.12033 24811882

[B35] Gibson J. J. (1966). *The Senses Considered as Perceptual Systems.* Boston: Houghton Mifflin.

[B36] Grimm S. R. (2006). Is understanding a species of knowing? *Br. J. Philos. Sci*. 57 315–535. 10.1093/bjps/axl015

[B37] Harnad S. (1990). The symbol-grounding problem. *Physica* 42 335–346. 10.1016/0167-2789(90)90087-6

[B38] Iran-Nejad A. (1978). *An Anatomic [embodied] Account of Knowing.* Master’s thesis equivalence paper, University of Illinois Champaign, IL.

[B39] Iran-Nejad A. (1980/1987). “The schema: a long-term memory structure or a transient functional pattern,” In *Understanding Reader’s Understanding* eds Tierney R. J. Anders J. N. (Hillsdale, NJ: Erlbaum) 109–128.

[B40] Iran-Nejad A. (1983a). Can cognitive activity directly influence the intensity of hedonic tone? *Paper Presented at the Annual Meeting of the Midwestern Psychological Association* Chicago, IL.

[B41] Iran-Nejad A. (1983b). *Qualitative and Quantitative Causes of the Experience of Affect.* Doctoral Dissertation, University of Illinois Urbana-Champaign, IL.

[B42] Iran-Nejad A. (1984). Qualitative and quantitative aspects of the comprehension of surprising information. *Paper Presented at the Annual Meeting of the Midwestern Psychological Association* Chicago, IL.

[B43] Iran-Nejad A. (1986). Understanding surprise-ending stories: long-term memory schemas versus schema-independent content elements. *J. Mind Behav.* 7 37–62.

[B44] Iran-Nejad A. (1987). Cognitive and affective causes of interest and liking. *J. Educ. Psychol.* 79 120–130. 10.1037/0022-0663.79.2.120 1786484

[B45] Iran-Nejad A. (1989a). Associative and nonassociative schema theories of learning. *Bull. Psychon. Soc.* 27 1–4. 10.3758/BF03329880

[B46] Iran-Nejad A. (1989b). A nonassociative schema theory of cognitive incompatibility. *Bull. Psychon. Soc.* 27 429–432. 10.3758/BF03334647

[B47] Iran-Nejad A. (1989c). A nonconnectionist schema theory of understanding surprise-ending stories. *Discourse Processes* 12 127–148. 10.1080/01638538909544723

[B48] Iran-Nejad A. (1990). Active and dynamic self-regulation of learning processes. *Rev. Educ. Res.* 60 573–602. 10.1016/j.apmr.2011.12.003 22289226

[B49] Iran-Nejad A. (2000). Knowledge, self-regulation, and the brain-mind cycle of reflection. *J. Mind Behav.* 21 67–88.

[B50] Iran-Nejad A. (2016). *A Whole Theme Cross-Disciplinary Organizer for the Four Biofunctional-Relevance Quadrants of Exemplary Science and Technology: Whole Theme Education Project.* Vienna: Online Biographical Bibliography (OBB).

[B51] Iran-Nejad A. Cecil C. (1992). “Interest and learning: a biofunctional perspective”, in *The Role of Interest in Learning and Development* eds Renninger K. A. Hidi S. Krapp A. (Hillsdale, NJ: Erlbaum) 297–332.

[B52] Iran-Nejad A. Chissom B. S. (1992). Contributions of active and dynamic self-regulation to learning. *Innov. High. Educ.* 17 125–136. 10.1007/BF00917134

[B53] Iran-Nejad A. Clore G. L. Vondruska R. I. (1981/1984). Affect: a functional perspective. *J. Mind Behav.* 5 279–310.

[B54] Iran-Nejad A. Gregg M. (2001). The brain-mind cycle of reflection. *Teach. Coll. Rec.* 103 868–895. 10.1111/0161-4681.00137

[B55] Iran-Nejad A. Gregg M. (2011). The nonsegmental context of segmental understanding: a biofunctional systems perspective. *Am. J. Educ. Stud.* 4 41–60.

[B56] Iran-Nejad A. Homaifar A. (2000). The nature of distributed learning and remembering. *J. Mind Behav.* 21 153–184.

[B57] Iran-Nejad A. Irannejad A. B. (2017a). Commentary: does mindfulness enhance critical thinking? Evidence for the mediating effects of executive functioning in the relationship between mindfulness and critical thinking. *Front. Educ. Educat. Psychol.* 8 10.3389/feduc.2017.00008 PMC4717844 26834669

[B58] Iran-Nejad A. Irannejad A. B. (2017b). Conceptual and biofunctional embodiment: a long story on the transience of the enduring mind. *Front. Psychol.* 7:1900. 10.3389/fpsyg.2016.01990 28119639 PMC5222851

[B59] Iran-Nejad A. Marsh G. E. Clements A. C. (1992). The figure and the ground of constructive brain functioning: beyond explicit memory processes. *Educ. Psychol.* 27 473–492. 10.1207/s15326985ep2704_5

[B60] Iran-Nejad A. Ortony A. (1982). *Cognition: A Functional View.* Rockville, MD: ERIC Clearinghouse

[B61] Iran-Nejad A. Ortony A. (1984). A biofunctional model of distributed mental content, mental structures, awareness, and attention. *J. Mind Behav.* 5 171–210.

[B62] Iran-Nejad A. Stewart W. Robinson C. (2015). First-person educational psychology for teacher education majors: a spontaneous biofunctional understanding intervention. *Int. J. Educ. Psychol.* 4 252–279. 10.17583/ijep.2015.896

[B63] Iran-Nejad A. Winsler A. (2000). Bartlett’s schema theory and modern accounts of learning and remembering. *J. Mind Behav.* 21 5–35.

[B64] Jenkins J. J. (1974). Remember that old theory of memory? Well, forget it. *Am. Psychol.* 29 785–795. 10.1037/h0037399

[B65] Jin Z. Lee Y. Yuan Z. (2016). Biofunctional understanding and judgment of size. *Front. Psychol.* 7:436. 10.3389/fpsyg.2016.00436 27047438 PMC4805645

[B66] Jin Z. Lee Y. Zhu J. (2015). Control your mind, make affordance available. *Front. Psychol.* 6. 10.3389/fpsyg.2015.00096 25741298 PMC4330679

[B67] Johnson M. (2015). Embodied understanding. *Front. Psychol. Cogn.* 6:875. 10.3389/fpsyg.2015.00875 26175701 PMC4484222

[B68] Khemlani S. S. Barbey A. K. Johnson-Laird P. N. (2014). Causal reasoning with mental models. *Front. Hum. Neurosci.* 8:849. 10.3389/fnhum.2014.00849 25389398 PMC4211462

[B69] Kiverstein J. (2012). The meaning of embodiment. *Top. Cogn. Sci.* 4 740–758. 10.1111/j.1756-8765.2012.01219.x 22893588

[B70] Lakoff G. Johnson M. (1980). Metaphors we live by 1st Edn. Chicago: University of Chicago Press.

[B71] McClelland J. L. Rumelhart D. E. (eds). (1986). “Parallel distributed processing: Explorations in the microstructure of cognition,” in *Psychological and Biological Models* Vol. 2 (Cambridge, MA: MIT Press) 122–169. 25087578

[B72] McCombs B. L. (1991). Overview: where have we been and where are we going in understanding human motivation? *J. Exp. Educ.* 60 5–14. 10.1080/00220973.1991.10806576

[B73] McCulloch C. E. Searle S. R. (2000). *General, Linear and Mixed Models.* New York, NY: Willey 10.1002/0471722073

[B74] Miller J. (1978). *The Body in Question.* New York, NY: Random House.

[B75] Neisser U. (1967). *Cognitive Psychology.* New York, NY: Appleton-Century-Crofts.

[B76] Neisser U. (1976). *Cognition and Reality: Principles and Implications of Cognitive Psychology.* San Francisco: WH Freeman.

[B77] Neisser U. ed. (1987). “From direct perception to conceptual structure,” *Concepts and Conceptual Development: Ecological and Intellectual Factors in Categorization* (New York, NY: Cambridge University Press) 11–24.

[B78] Neisser U. (1994). Multiple systems: a new approach to cognitive theory. *Eur. J. Cogn. Psychol.* 6 225–241. 10.1080/09541449408520146

[B79] Neisser U. Jopling D. A. (1997). *The Conceptual Self in Context: Culture, Experience, Self-Understanding.* Cambridge: Cambridge University Press.

[B80] Neisser U. Winograd E. (1988). *Remembering Reconsidered: Ecological and Traditional Approaches to the Study of Memory.* Cambridge: Cambridge University Press 193–243. 10.1017/CBO9780511664014

[B81] Noone C. Bunting B. Hogan M. J. (2016). Does mindfulness enhance critical thinking? Evidence for the mediating effects of executive functioning in the relationship between mindfulness and critical thinking. *Front. Psychol. Cogn.* 6:2043. 10.3389/fpsyg.2015.02043 26834669 PMC4717844

[B82] Posner G. J. Strike K. A. Hewson P. W. Gertzog W. A. (1982). Accommodation of a scientific conception: towards a theory of conceptual change. *Sci. Educ.* 66 211–227. 10.1002/sce.3730660207

[B83] Prawat R. S. (2000). Keep the solution, broaden the problem: commentary on ”knowledge, self-regulation, and the brain-mind cycle of reflection”. *J. Mind Behav.* 21 89–96.

[B84] Roediger H. L. (1980). Memory metaphors in cognitive psychology. *Mem. Cognit.* 8 231–246. 10.3758/BF03197611 7392950

[B85] Rosch E. (2000). The brain between two paradigms: can biofunctionalism join wisdom intuitions to analytic science. *J. Mind Behav.* 21 189–203.

[B86] Rosch E. (2001). If your depict a bird, give it space to fly”: eastern psychologies, the arts, and self-knowledge. *SubStance* 30 236–253. 10.2307/3685515

[B87] Rumelhart D. E. (1975). “Notes on a schema for stories,” in *Representation and Understanding: Studies in Cognitive Science* eds Bobrow D. G. Collins A. (New York, NY: Academic Press) 211–236.

[B88] Rumelhart D. E. (1976). “Toward an interactive model of reading,” in *Attention and performance* Vol. 6 ed. Dornic S. (London: Academic Press).

[B89] Rumelhart D. E. (1980). “Schemata: the building blocks of cognition,” in *Theoretical Issues in Reading Comprehension* eds Spiro R. J. Bruce B. C. Brewer W. F. (Hillsdale, NJ: Erlbaum) 33–58.

[B90] Rumelhart D. E. (1984). “The emergence of cognition from subsymbolic processes,” in *Proceedings of the Sixth Annual Conference of the Cognitive Science Society* Boulder, CO 59–62.

[B91] Rumelhart D. E. Hinton G. E. McClelland J. L. (1986). “A general framework for parallel distributed processing,” in *Parallel Distributed Processing: Explorations in the Microstructure of Cognition* Vol. 1 eds Rumelhart D. E. McClelland J. L. (Cambridge, MA: The MIT Press).

[B92] Rumelhart D. E. McClelland J. L. (eds). (1986). “Learning internal representations by error propagation,” in *Parallel Distributed processing: Explorations in the microstructure of cognition* Vol. 1 Cambridge, MA: MIT Press

[B93] Rumelhart D. E. Ortony A. (1977). “The representation of knowledge in memory,” in *Schooling and the Acquisition of Knowledge* eds Anderson R. C. Spiro R. J. Montague W. E. (Hillsdale, N.J.: Erlbaum) 99–135.

[B94] Ryle G. (1949). The concept of mind. New York, NY: Hutchinson.

[B95] Salomon G. (2006). “The systemic vs. analytic study of complex learning environments,” in *Handling Complexity in Learning Environments: Theory and Research* eds Elen J. Clark R. E. Lowyck J. (Boston: Elsevier) 255–264.

[B96] Scott D. S. (1990). “The computational conception of mind: a panel discussion,” in *Acting and rflecting (Synthese Libray): The Interdisciplinary turn in philosophy* ed. Sieg W. (Dordrecht: Kluver Academic Publishing) 39–56.

[B97] Schallert D. L. (1982). “The significance of knowledge: a synthesis of research related to schema theory,” in *Reading Expository Material* ed. Otto W. (New York, NY: Elsevier) 13–47.

[B98] Schneider W. Shiffrin R. M. (1977). Controlled and automatic human information processing: I. Detection, search, and attention. *Psychol. Rev.* 84 1–66. 10.1037/0033-1295X.1084.1031.1031

[B99] Schön D. A. (1983). *The Reflective Practitioner: How Professionals Think in Action.* New York, NY: Basic Books.

[B100] Scorolli C. Borghi A. M. (2015). Square bananas, blue horses: the relative weight of shape and color in concept recognition and representation. *Front. Psychol.* 6:1542. 10.3389/fpsyg.2015.01542 26500593 PMC4597035

[B101] Shiffrin R. M. Schneider W. (1977). Controlled and automatic human information processing: II. Perceptual learning, automatic attending, and a general theory. *Psychol. Rev.* 84 127–190. 10.1037/0033-295X.84.2.127

[B102] Shulman L. S. (1984). The practical and the eclectic: a deliberation on teaching and educational research. *Curriculum Inq.* 14 183–200. 10.1080/03626784.1984.11075920

[B103] Shulman L. S. (1986). Those who understand: knowledge growth in teaching. *Educ. Res.* 15 4–14. 10.3102/0013189X015002004

[B104] Shulman L. S. (1991). Ways of seeing, ways of knowing: ways of teaching, ways of learning about teaching. *J. Curriculum Stud.* 23 393–395. 10.1080/0022027910230501

[B105] Shulman L. S. (1999). What is learning and what does it look like when it doesn’t go well? *Change* 31 10–17. 10.1080/00091389909602695

[B106] Shulman L. S. (2002). Making differences: a table of learning. *Change* 34 36–44. 10.1080/00091380209605567

[B107] Smolensky P. (1987). Connectionist AI, symbolic AI, and the brain. *Artif. Intell. Rev.* 1 95–109. 10.1007/BF00130011

[B108] Soylu F. (2016). An embodied approach to understanding: Making sense of the world through simulated bodily activity. *Front. Psychol.* 7:1914. 10.3389/fpsyg.2016.01914 27999558 PMC5138236

[B109] Thill S. Twomey K. E. (2016). What’s on the inside counts: a grounded account of concept acquisition and development. *Front. Psychol.* 7:402. 10.3389/fpsyg.2016.00402 27047427 PMC4804724

[B110] Thorndyke P. W. Yekovich F. R. (1980). A critique of schema-based theories of human story memory. *Poetics* 9 23–49. 10.1016/0304-422X(80)90011-X

[B111] Thurmond P. J. (1978). If cornered, scream. *Ellery Queens Mystery Mag.* 71 66–68.

[B112] Varela F. J. Thompson E. Rosch E. (1991). *The Embodied Mind: Cognitive Science and Human Experience.* Cambridge, MA: MIT Press.

[B113] Wilensky R. (1983). Story grammars versus story points. *Behav. Brain Sci.* 6 579–623. 10.1017/S0140525X00017520

[B114] Williams J. N. (2008). Propositional knowledge and know-how. *Synthese* 165 107–125. 10.1007/s11229-007-9242-1

[B115] Willis J. W. (2000). Defining a field: content, theory, and research issues. *Contemp. Issues Technol. Teach. Educ.* 1 209–219. 26269785

[B116] Wilson M. (2002). Six views of embodied cognition. *Psychon. Bull. Rev.* 9 625–636. 10.3758/BF03196322 12613670

[B117] Ziemke T. Bergfeldt N. Buason G. Susi T. Svensson H. (2004). Evolving cognitive scaffolding and environment adaptation: a new research direction for evolutionary robotics. *Conn. Sci.* 16 339–350. 10.1080/09540090412331314821

